# Dental Hygiene Challenges in Children with Autism: Correlation with Parental Stress: A Scoping Review

**DOI:** 10.3390/jcm13164675

**Published:** 2024-08-09

**Authors:** Pablo López Alegría, Síbila Floriano Landim, Braulio Henrique Magnani Branco, Florencia Carmine, Katherine Birditt, Cristian Sandoval, Manuel Martín González

**Affiliations:** 1 Escuela de Terapia Ocupacional, Facultad de Psicología, Universidad de Talca, Talca 3465548, Chile; pablo.lopez@utalca.cl (P.L.A.); sibila.floriano@utalca.cl (S.F.L.); 2 Graduate Program in Health Promotion, Cesumar University (UniCesumar), Maringá 87050-900, Brazil; braulio.branco@unicesumar.edu.br; 3 Carrera de Medicina, Facultad de Medicina, Universidad de La Frontera, Temuco 4811230, Chile; f.carmine02@ufromail.cl; 4 Physiology Development and Neuroscience Department, University of Cambridge, Cambridge CB2 1TN, UK; krb56@cam.ac.uk; 5 Escuela de Tecnología Médica, Facultad de Salud, Universidad Santo Tomás, Los Carreras 753, Osorno 5310431, Chile; 6 Departamento de Medicina Interna, Facultad de Medicina, Universidad de La Frontera, Temuco 4811230, Chile; 7 Núcleo Científico y Tecnológico en Biorecursos (BIOREN), Universidad de La Frontera, Temuco 4811230, Chile; 8 Hospital Universitario Torrecárdenas, 04009 Almería, Spain

**Keywords:** autism spectrum disorder, dental hygiene, oral health, stress, scoping review

## Abstract

**Background**: Children diagnosed with autism spectrum disorders are shown to have poor periodontal health and dental hygiene habits. Extensive research has revealed that parents of children with autism spectrum disorder (ASD) frequently encounter heightened levels of stress, despair, and anxiety in comparison to parents of neurotypical children. The aim was to understand the relationship between the dental hygiene of children with ASD and the stress generated in their parents. **Methods**: A scoping review was carried out to identify any gaps or research opportunities for clinical practice concerning oral care and stress levels in parents in the PubMed, Medline, ScienceDirect, and Scopus databases. **Results**: A total of 139 articles were reviewed. Of these, only 10 met the selection criteria for inclusion. Our results reveal a lack of studies presenting evidence on the topic of poor dental hygiene in children with ASD and high stress levels in their parents. **Discussion**: There is ample evidence that children with ASD have poor dental hygiene, as well as higher levels of stress in their parents. However, little or no evidence links these two variables. Future studies should focus on this link, which could have practical implications for improving dental care for children with ASD.

## 1. Introduction

Autism, recognized within the clinical sense as autistic spectrum disorder (ASD), is a complex condition characterized by neurological alterations that arise in early infancy, leading to deficits in communication and social interaction, and manifestations of repetitive and restricted behavior [1,2,3,4]. According to the World Health Organization [5], approximately 1 in 100 children is diagnosed with autism, which motivates health authorities and the scientific community to conduct research and develop policies aimed at caring for this population and its family environment.

Concerning the health consequences of ASD for child populations, there is a notably high risk of oral pathologies due to a lack of oral hygiene. This shortfall arises from the limited capacity, abilities, and psychomotor skills that keep children with ASD from carrying out adequate oral hygiene, leading to higher odds of developing oral habits [6,7,8]. Despite previous studies addressing oral health issues in autistic children and stress levels among their parents, the current study takes a more comprehensive approach.

Consequently, the hypothesis that arises from an initial exploration refers to the fact that there is a knowledge gap in the literature about the level of stress among parents of children with ASD who have poor dental hygiene.

One of the most notable studies dealing with the problem of oral health among children with ASD was conducted by Alqahtani et al. [9], whose objective was to evaluate the knowledge and attitudes of the parents of autistic children regarding oral health and the oral hygiene habits of their children. To this end, the researchers conducted a survey with parents in various care centers. Overall, the majority of parents who were consulted collaborated on the oral hygiene practices of their autistic children. However, there was no consideration of the stress levels that parents faced in this situation.

AlHumaid [10] conducted a pertinent literature review on the dental health experiences of children with ASD in Saudi Arabia. The results revealed a lack of studies and publications in Arabia, which could have affected the limited development of effective oral health policies. In another review article, Pimienta et al. [11] also emphasized the importance of early detection of ASD for timely treatment, particularly concerning orthodontic care and oral health in general.

Along the same lines, Erwin et al. [12] conclude that diverse factors affected both the adoption of healthy dental hygiene behaviors and access to orthodontic care for autistic children and adolescents. These included factors directly related to their ASD diagnosis, such as a lack of communication, and other commonly associated elements of autism, such as deficits in sensitivity and sensory perception. This finding meshes with Stein et al. [13], who reported on the high levels of behavioral stress and anxiety among autistic children during routine dental visits, compared with a typical group.

Bernath and Kanji [1] mentioned that people with ASD had a wider range of ailments and faced greater oral care barriers. Their principal findings included the identification of key themes, including care barriers, inhibited social and communication skills, dependency on parents, the clinical environment, and oral health professionals’ skills for treating clients with special-care needs.

Como et al. [2] mentioned that children with ASD faced the risk of suffering oral health ailments and analyzed the reasons why this occurred in their study. They also proposed interprofessional collaboration between dentists and occupational therapists to provide sensitization strategies before clinical visits and to help children with ASD to regulate their emotions during clinical treatments. Similarly, Mohamed and Abdalla [14] proposed a behavioral orientation focus in order to provide dental care to patients with ASD, while Nunes da Silva et al. [15], in a systematic review and meta-analysis, examined the state of oral health for children and youths with ASD.

Park et al. [16] analyzed the concept of dental anxiety, indicating that it was greater in children with ASD due to its association with feelings of impotence, loss of control, and sensory overload. The results reflect high dental anxiety in 68% of participants, with fear of pain causing the greatest concern. The authors generally observed no significant correlations between dental anxiety and other variables of interest, including general anxiety severity, ASD symptoms, and symptoms of internalization and externalization, as well as sensory sensitivity. However, their findings can be useful for adapting treatments aimed at reducing dental anxiety in children with ASD. Finally, Tirado-Amador et al. [17] conducted a review of oral health in autistic people. They found that there was not a lot of evidence that showed how inadequate oral hygiene was in the homes of ASD patients or that they had cavities or other oral problems.

In terms of discussion, there is a consensus among researchers in the area regarding the limitations of children with ASD in achieving proper dental hygiene, caused by their typical behaviors of isolation; lack of communication; and deficits in attention, sensitiveness, and sensory perception [1,2,6,8,12]. Park et al. [16] also detected high dental anxiety levels among children with ASD, probably related to sensations of incapacity, lack of control, and overstimulation, which align with Stein et al. [13] concerning heightened anxiety levels.

Many studies agreed that the dentist’s role in treating children with ASD was an important one [1,2,17], while other studies agreed that parents’ actions, especially their fears and how much they knew about how to handle the situation, were also important [9,17]. Finally, the evidence reported in the cited studies confirms the difficulty for autistic children to maintain adequate oral hygiene in order to reduce the odds of oral pathologies.

The studies generally show that parents of children with ASD report high stress levels, mainly associated with the special condition of their children [18,19,20,21,22,23,24]. Among the more recent studies, the most notable is Curley et al. [25], based upon a systematic review of stress reduction interventions for parents of children with ASD. Overall, the evidence proposes an integrative focus (mindfulness-based positive behavior support), which showed greater effectiveness in reducing parental stress.

A systematic review carried out analyzed the association between parents’ stress and the positive re-evaluation of coping and quality of life for parents of children with ASD [26]. This review reflected how parents of autistic children presented high stress levels, associated with the ineffective use of coping strategies and a generally low quality of life. Along the same lines, Ng et al. [27] presented another systematic review about stress among parents of children with ASD, but specifically in China.

In another literature review, Jolly and Huber [28] examined documents indicating high rates of anxiety and stress, specifically among mothers of children with ASD. Dolev et al. [29] evaluated 45 preschool-age children with ASD and their mothers using emotional-availability scales. Both studies observed a consistent pattern of high anxiety and stress levels among mothers in the analyzed studies that was attributed to the unique condition of their autistic children. Finally, May et al. [30] also explored the relations between the quality of co-parenting, the specific parental self-efficacy of autism, and parental stress in the mothers and fathers of children with ASD. Their findings suggest that both mothers and fathers reported similarly high stress levels related to co-parenting quality and autism-specific parental self-efficacy. The results also reflected the importance of considering the specific role of parental emotional regulation, family functioning, and educational level, which influence maternal stress and the suffering of partnered parents. Therefore, the present article aims to identify the gaps or lacunae in scientific research regarding the topic of dental hygiene in children with autism and their parents’ stress.

## 2. Material and Methods

A scoping review methodology was used, which is a systematic and rigorous approach to methodically exploring a set of relevant documents, including peer-reviewed articles, research book chapters, and presentations at scientific events, regarding a specific topic. The main objective was to provide a “map” of available evidence and indicate any knowledge gaps [31].

A scoping review was conducted because our initial exploration of the literature revealed a clear lack of empirical work or original research that provided data on the raised topic. On the one hand, this initial condition impedes systematic reviews and meta-analyses, which rely heavily on evidence from primary or original results and data. However, an initial exploration revealed a research gap, prompting the need for a scoping review to objectively confirm the lack of original research on the stress levels of parents of children with ASD who maintain poor dental hygiene.

The central procedure consisted of seeking and analyzing scientific articles from the main medical literature databases—PubMed, Medline, ScienceDirect, and Scopus—complying with the **PICoR** standard: **P** = participants, **I** = intervention, **C** = comparison, and **R** = results (outcomes). In addition, the following inclusion and exclusion criteria were considered for the final selection of the articles under analysis:

The following inclusion and exclusion criteria were applied to the articles under analysis:

### 2.1. Inclusion Criteria

The search included empirical research articles and evidence-based reviews, solely in English, and including at least two of the three study target elements or variables: dental hygiene, ASD in children, and stress in parents of children with ASD.

### 2.2. Exclusion Criteria

The search excluded systematic and narrative review articles, articles in languages other than English, articles that included only one of the three analysis elements, and papers not indexed in the selected databases.

### 2.3. Search Period

The search period began in June 2023 and ended in December 2023, encompassing articles published between 2010 and 2023.

### 2.4. Search Formulae

After entering the selected databases, the following keywords were entered with their respective Boolean operators. The search was performed during the months of June and July 2023. Specifically, the search equations and respective combinations were as follows: “dental hygiene” AND “children with autism”, “dental hygiene” AND “children with ASD”, “oral health” AND “children with autism”, “oral health” AND “children with ASD”, “stress in parents” AND “children with autism”, “stress in parents” AND “children with ASD”, “anxiety in parents” AND “children with autism”, “anxiety in parents” AND “children with ASD”, “depression in parents” AND “children with autism”, “depression in parents” AND “children with ASD”, “dental hygiene” OR “oral health” AND “children with Autism Spectrum Disorder (ASD)” AND “stress in parents”, “dental hygiene” OR “oral health” AND “children with autism” AND “stress in parents”, “dental hygiene” OR “oral health” AND “children with ASD” AND “stress in parents”, “dental hygiene” OR “oral health” AND “children with autism” AND “anxiety in parents”, “dental hygiene” OR “oral health” AND “children with ASD” AND “anxiety in parents”, “dental hygiene” OR “oral health” AND “children with autism” AND “depression in parents”, and “dental hygiene” OR “oral health” AND “children with ASD” AND “depression in parents”.

### 2.5. Step-by-Step Procedure

**Figure 1:**
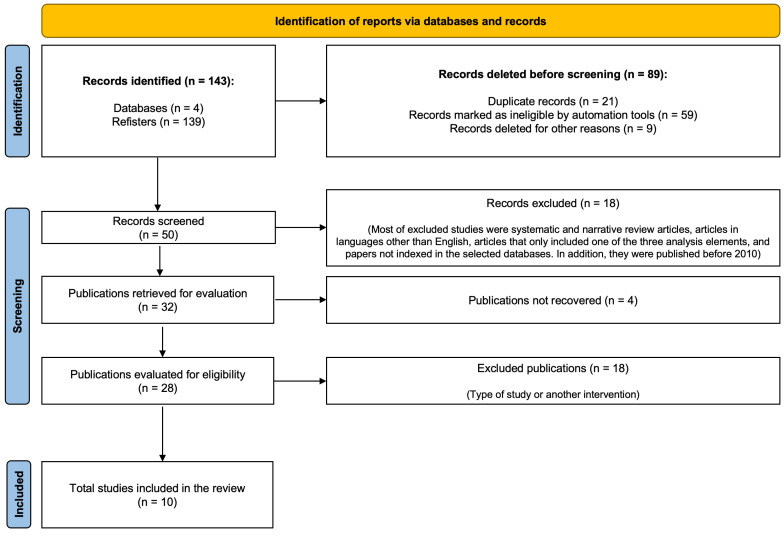
Step-by-step article-selection procedure.

Figure 1 graphically illustrates the step-by-step procedure in the next section.

1. Identification of Records in Each Database (PubMed, Medline, ScienceDirect, and Scopus) via Search Formulae
2. Exclusion of Matching or Duplicate Records between Databases
3. Record Selection by Title and Abstract, with Inclusion Criteria
4. Review and Evaluation of Complete Articles, Including Principal Findings
5. Final Selection of Articles for Analysis

The co-authors of the article carried out and verified these steps to ensure the consistency and reliability of the obtained results. Figure 1 provides a graphic representation of these steps.

### 2.6. Procedure for Analyzing the Selected Articles

After completing the screening and final selection process for the articles, the following analysis was conducted:

#### 2.6.1. PICO Standard

Following the PICO standard, the articles were tabulated based on the following fields: authors and year of publication, participants, intervention, comparison, and results (outcomes).

#### 2.6.2. Abstract in Each Field

A concise abstract was made for each article within the aforementioned fields.

#### 2.6.3. Qualitative Analysis 

The analytical focus was primarily qualitative, centered on identifying the main topics addressed. The goal was to obtain the evidence necessary to thoroughly approach the research question.

#### 2.6.4. Contrast between Articles 

The selected articles were compared, considering both the theme and the outcomes of each study.

#### 2.6.5. Discussion of Thematic Connections

The discussion was centered on describing the thematic connections between the analyzed articles. The coincidences, differences, and contradictions in the results observed between these articles and those reported in the background of the present study were highlighted.

## 3. Results

A total of 10 articles were identified and selected for analysis based on the inclusion criteria established as of October 2023. Despite conducting an exhaustive search, no studies that simultaneously addressed all three variables of interest were found: dental hygiene or oral health, ASD in children, and stress in parents of autistic children. However, all the articles selected fulfilled the inclusion criteria by being original (empirical) studies that considered at least two of the relevant variables.

**Figure 2:**
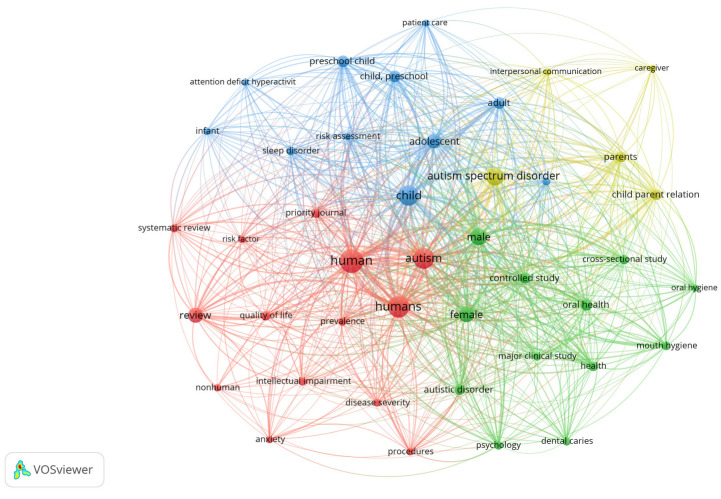
Topical distribution of the literature on dental hygiene and oral health among autistic children.

**Figure 3:**
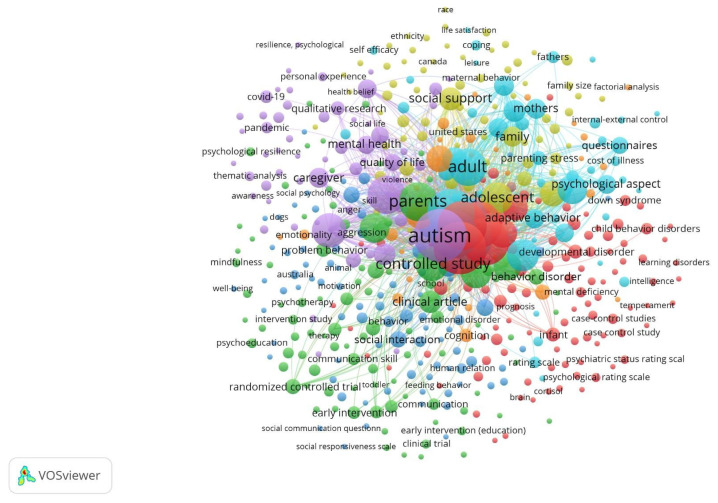
The literature on stress levels among autistic children’s parents.

Of the 10 articles selected, 8 focused specifically on the topic of dental hygiene and oral health in autistic children, while only 2 addressed the topic of stress levels in parents of children with ASD. Table 1 summarizes the evidence provided by these articles in relation to the research questions and PICO standards. Ten of the analyzed studies, published between 2020 and 2023, suggest a relatively short “half-life” or “semi-period” and the contemporary nature of the reviewed literature. The database with the largest number of records was Scopus. Using this information, a thematic distribution map using “VOSviewer version 1.6.20” software was generated. Figure 2 shows the specific distribution of topics related to dental hygiene and autistic children, while Figure 3 shows the clustering of the literature about stress levels in autistic parents. The search generated both figures based on the keywords and their combinations.

Despite the detailed graphic representation, no articles appeared that addressed studies on all three of the aforementioned variables: oral health or dental hygiene, autism in children, and stress in parents of autistic children. This lack of overlap suggests a knowledge gap in the specialized literature. In this context, a research gap is defined as a scientific question without any responses within academic circles, providing an opportunity to present a publishable article given its novelty, originality, and unique character [39].

Figure 2 illustrates how the topic of dental hygiene divides the literature, with the blue nodes representing the topic and the green nodes representing their intersection. The topic of autism in children starts with the red nodes and then fuses with the green nodes, signifying a strong thematic association between these two variables. The size of the central nodes (in green) also confirms the existence of a set of studies where children with ASD were the primary topic and oral health was a secondary associated theme. This graphic report is consistent with the eight records on the aforementioned topics.

The distribution in Figure 3 resembles the previous one in the sense that the main topic is still autism, represented in the violet-colored central node. In this case, though, the secondary associated topic is parental stress, represented in smaller sky-blue nodes. An especially notable element is the broad dispersion of nodes in various linked topics, while in the central nodes, there is no appreciable fusion between the topics of autism in children and parental stress. This aligns with the previously mentioned finding that there were a smaller number of studies about stress levels in the parents of autistic children (four articles) compared with the number of studies associating dental hygiene with children with ASD (eight articles).

## 4. Discussion

### 4.1. Summary of Key Findings and Interpretation

In general terms, the results derived from analyzing the articles reveal some convergences and divergences with prior studies on the same topic. When specifically focusing on oral health in children with ASD, AlHumaid et al. [32] concluded that deficient oral health in these children was not associated with parents’ attitudes, indicating that the limitation in their oral health is inherent to their autism and not the result of any lack of parental attention. This finding aligns with the results from Permatasari et al. [36], who found no correlation between the knowledge, attitudes, and oral health practices of mothers and the prevalence of cavities in their children with autism.

By contrast, Alqahtani et al. [9] report on a positive dental hygiene practice by parents toward their autistic children, highlighting significant parental participation in this task. These results support the observations by Omer [40], who reported that two-thirds of parents provided support during tooth brushing, and Parry et al. [35], whose study revealed parental knowledge about the importance of positive oral health messages and the need to encourage dental hygiene habits in their autistic children. In summary, Verma et al. [38] confirmed divergent perceptions about oral health between the parents of autistic children and the parents of allistic children.

The discrepancy between the findings from AlHumaid et al. [32] and Alqahtani et al. [9] highlights the importance of considering the methodological focus. While the former found no relation between parents’ attitudes regarding the oral health of their autistic children, the latter stressed positive care from parents for the dental hygiene of their children with ASD. Specifically, AlHumaid et al. [32] conducted a correlation analysis using the Pearson correlation coefficient, while Alqahtani et al. [9] applied a survey to a sample of parents of children with ASD, resulting in a percentual distribution analysis.

Building on the preceding point, Farias et al. [3] used a sample of parents of children with ASD to analyze some variables, including brushing frequency, bleeding gums, and “dental crunching”, to conclude that these factors predict more negative parental perceptions regarding the oral health of their autistic children, in contrast with the results from Parry et al. [35]. Another notable study was conducted by Prakash et al. [37] regarding parents’ perceptions about oral health and quality of life for autistic children, which concluded that the association between autism and oral health effects negatively impacted quality of life for both autistic children and their parents.

Alibekova et al. [33] conducted an analysis of the stress and anxiety levels of parents of children with autism spectrum disorder (ASD) in relation to their material conditions. They found that parents with unmet basic needs and whose financial constraints prevent them from affording clinical care services for their autistic children experienced higher levels of stress. The aforementioned study is related to the work by Curley et al. [25], who, given the evidence on the high stress level among the parents of autistic children, proposed intervention programs with an integrative focus (Mindfulness-Based Positive Behavior Support).

On the other hand, high levels of perceived stress may be associated with changes in muscle asymmetry, particularly in the act of clenching the teeth and bruxism [41]. Likewise, anxiety and depression are strongly associated with migraines and tension headaches [42]. Other variables that manifest as negative effects of stress in parents at a physiological level are the increase in heart rate and high levels of the hormone cortisol [34].

There are also parallels to the study by Enea and Rusu [43], who found that maladaptive behaviors and perception and sensitivity deficits in autistic children are the strongest parental stress indicators, manifesting most strongly with single mothers. Another study specifically focused on the parents of autistic children came from Foody et al. [35]; the study aligns with the studies by Alibekova et al. [33], Curley et al. [25], and Enea and Rusu [43], given that it reports high stress and anxiety levels in the parents of autistic children. However, Foody et al. [34] found that, despite the high stress and anxiety levels, anxiety levels were moderate.

### 4.2. Scoping and Limitations

There is a notable disparity in the records between the various databases consulted. This variability led to a search with low concentration, generating significant differences in the number of articles available in each database. This disparity could have introduced biases in study selection and affected the representativeness of the evidence gathered. However, this limitation is acknowledged and warned to readers so that future research can take it into account.

The second limitation was the scarcity of studies that presented empirical evidence from primary sources, as opposed to those that focused on literature reviews. The prevalence of review studies could affect the robustness of the empirical grounds of the review, ultimately compromising the solidity of the evidence-based conclusions. However, the existing research gap in the available scientific literature was highlighted to describe the findings as objectively as possible. This trend also highlights the need for more original studies directly addressing the relationship between autistic children’s dental hygiene and their parents’ stress levels.

### 4.3. Future Studies

Our findings prompt us to recommend further research on this specific topic to confirm the hypothesis that dental hygiene in autistic children contributes to their parents’ stress levels. Future studies, in addition to filling the existing research gap and testing the aforementioned hypothesis, can promote programs and actions aimed at developing dental hygiene skills in autistic children, as well as strategies to reduce parents’ stress levels.

It is recommended to design and validate a scale to gauge the stress levels of parents of children with ASD, particularly when they have poor dental hygiene and are at risk of oral pathologies. The application of said instrument could provide valuable data and information for the care and implementation of programs aimed at families of autistic children.

Additionally, a series of diagnoses must be carried out on the parents’ cognitive and volitional abilities to face situations of high levels of stress generated by the exceptional condition of their children with ASD in order to apply information programs on what to do in certain situations.

It is also recommended that researchers study other major stressors for parents of autistic children, including the communication deficit in these children, their repetitive behaviors, and oral health. This would generally help improve the quality of life for autistic children and their family environments.

Equally important is research into dentists’ specialized training to care for this unique population of autistic children. Such studies could determine the profile of instrumental and interpersonal skills required by the dental professional to care not only for children with ASD but also to provide peace of mind to their parents.

It is also suggesting a bibliometric analysis of production regarding the topic of dental hygiene in children with ASD and their parents’ stress levels in order to observe trends, publication impact, the most productive authors, and collaboration networks.

## 5. Conclusions

Since no published studies address the specific research question on the relationship between dental hygiene in autistic children and their parents’ stress levels, our exhaustive review reveals a significant scientific-research gap. It is evident that existing studies have primarily centered on independently analyzing topics, such as “dental hygiene or oral health in autistic children” or “stress levels in the parents of autistic children”.

More articles on the topic of “dental hygiene or oral health in autistic children” than on the topic of “stress levels in parents of autistic children” were selected and analyzed. Based on this, one could argue that studies on dental hygiene among autistic children have produced more research than those on parental stress and anxiety, but further validation through a bibliometric study is necessary.

An element associated with the integral research question was identified based on the evidence found. This finding refers to the study by Prakash et al. [37], who reported on how oral health affections among autistic children negatively affected their parents’ quality of life. However, it should be noted that these results do not specifically address parents’ stress or anxiety levels, thus limiting their usefulness in answering our research question and leaving a research gap.

Overall, the review results empirically support the high prevalence of oral ailments in autistic children, attributed to the limitations in skill and habit-forming needed for proper oral hygiene. Extant studies also confirm the presence of high stress levels in the parents of autistic children who have high stress levels due to the traits and limitations imposed by ASD on their children.

Finally, there is a growing need to carry out specific studies examining parental stress levels related to their autistic children’s oral hygiene; a factor that could heighten their stress must be highlighted. This would not only help close the research gap identified herein but also offer valuable contributions to preserving parents’ mental health in this particular context.

## Figures and Tables

**Table 1:** Summary of evidence found in articles.

References	Participants	Intervention	Comparison	Results
[3]	There were 101 parents of children and adolescents without ASD and 100 parents of children and adolescents with ASD.	Quiz app	Two groups of parents were compared.	Variables such as brushing frequency, gum bleeding, and “dental gnashing” are predictors of the “worst” perception of parents in relation to the oral health of their autistic children.
[9]	There were 206 parents of children with ASD, including 77 fathers and 129 mothers.	Quiz app	The study compares the involvement of parents in their children’s activities.	The transmission of oral hygiene habits by parents was positive, and the majority of them participated in their autistic children’s dental hygiene practices.
[13]	A total of 44 children (*n* = 22 typical; *n* = 22 with ASD) aged 6–12 received routine dental cleanings.	Measurement of children’s communication skills, anxiety, and sensory processing.	The study variables allowed for a comparison between the group of children with ASD and the group with typical development.	Children with ASD show significantly greater uncooperativeness during routine dental cleanings compared to typically developing kids.
[32]	75 children with ASD.	A clinical evaluation of caries, gingivitis, and dental plaque was performed.	Parents’ attitudes regarding dental health were compared.	Parental attitudes toward dental care are not associated with autistic children’s dental health status.
[33]	146 parents of children with ASD.	A cross-sectional study examined the prevalence of stress, anxiety, and depressive symptoms.	The variables were compared according to the level of parental needs.	Parents with unmet needs reported higher levels of stress and anxiety, while those with met needs reported lower levels.
[34]	There were 80 mothers of children with ASD who received an independent diagnosis.	Quiz app: (1) cardiovascular evaluation, (2) saliva analysis, and (3) analysis of cortisol levels.	The analyzed variables were compared.	ASD mothers showed high levels of stress and anxiety but moderate levels of depression.
[35]	Does not specify.	Organizing a focus group with parents of ASD children.	Parents of autistic children in primary school and those of autistic children in secondary school form distinct groups.	Parents recognize the importance of positive messages regarding their autistic children’s oral health and the need to establish dental hygiene habits.
[36]	Thirty-four parents of children with ASD.	An observational analytical study using a cross-sectional research design was conducted.	Groups of mothers based on their knowledge, attitudes, and practices were compared.	The results reveal that there is no significant relationship between knowledge, attitudes, and practice in preserving oral health and the rate of caries and dental care needs in children with autism.
[37]	Three-hundred parents of children with ASD.	A self-assessment questionnaire was administered.	The quality of life of parents and children with ASD was compared.	The association between autism and oral health problems has a negative impact on both autistic children and their parents’ quality of life.
[38]	Parents of 154 ASD children and 235 normal children.	Groups of parents of ASD children and parents of normal children were compared.	Questionnaire.	The results show significant differences in the perception of oral health between parents of autistic children compared to parents of children without this condition.

ASD: autism spectrum disorder.

## Data Availability

The authors confirm that the data supporting the findings of this study are available within the article.

## References

[1] Bernath B., Kanji Z. (2021). Exploring barriers to oral health care experienced by individuals living with autism spectrum disorder. Can. J. Dent. Hyg..

[2] Como D.H., Stein Duker L.I., Polido J.C., Cermak S.A. (2021). Oral Health and Autism Spectrum Disorders: A Unique Collaboration between Dentistry and Occupational Therapy. Int. J. Environ. Res. Public Health.

[3] Farias A.C., Barbosa T., Duarte M. (2023). Parental Perception of the Oral Health-Related Quality of Life of Children and Adolescents with Autism Spectrum Disorder (ASD). Int. J. Environ. Res. Public Health.

[4] Fernández M.P., Espinoza A.E. (2019). Salud mental e intervenciones para padres de niños con trastorno del espectro autista: Una revisión narrativa y la relevancia de esta temática en Chile. Rev. Psicol..

[5] World Health Organization (2023). Autism: Key Facts.

[6] El Khatib A., El Tekeya M., El Tantawi M., Omar T. (2013). Oral health status and behaviours of children with Autism Spectrum Disorder: A case-control study. Int. J. Paediatr. Dent..

[7] Ferrer-Coba S., Martínez-Hernández N.L., Recio-Díaz T., Ferrer-Coba O. (2022). Atención estomatológica integral a niños con trastorno del espectro autista. Rev. Cienc. Méd. Pinar Río.

[8] Medina-Oropeza D., Rueda-Ventura M., Ramírez-Mendoza J., Hernández Abreu K. (2018). Cuidados bucodentales que tienen los padres con el estado de salud dental de los niños con Trastorno del Espectro del Autismo en el CRIAT. Rev. Tamé.

[9] Alqahtani A.S., Gufran K., Alsakr A., Alnufaiy B., Al Ghwainem A., Bin Khames Y.M., Althani R.A., Almuthaybiri S.M. (2023). Oral Healthcare Practices and Awareness among the Parents of Autism Spectrum Disorder Children: A Multi-Center Study. Children.

[10] AlHumaid J. (2022). Dental experiences related to oral care of children with autism spectrum disorders in Saudi Arabia: A literature review. Saudi Dent. J..

[11] Pimienta N., González Y., Rodríguez L. (2017). Autismo infantil, manejo en la Especialidad de Odontología. Acta Méd. Centro.

[12] Erwin J., Paisi M., Neill S., Burns L., Vassallo L., Nelder A., Facenfield J., Devalia U., Vassallo T., Witton R. (2022). Factors influencing oral health behaviours, access and delivery of dental care for autistic children and adolescents: A mixed-methods systematic review. Health Expect..

[13] Stein L.I., Lane C.J., Williams M.E., Dawson M.E., Polido J.C., Cermak S.A. (2014). Physiological and Behavioral Stress and Anxiety in Children with Autism Spectrum Disorders during Routine Oral Care. BioMed Res. Int..

[14] Mohamed H., Abdalla E. (2022). Behavioral Guidance Approaches to Provide Dental Care for Patients with Autism Spectrum Disorder: A Review of the Literature. Acta Sci. Dent. Sci..

[15] Nunes Da Silva S., Gimenez T., Souza R., Mello-Moura A., Raggio D., Morimoto S., Lara J.S., Soares G.C., Tedesco T.K. (2016). Oral health status of children and young adults with autism spectrum disorders: Systematic review and meta-analysis. Int. J. Paediatr. Dent..

[16] Park Y., Guzick A.G., Schneider S.C., Fuselier M., Wood J.J., Kerns C.M., Kendall P.C., Storch E.A. (2022). Dental Anxiety in Children with Autism Spectrum Disorder: Understanding Frequency and Associated Variables. Front. Psychiatry.

[17] Tirado-Amador L., Madera M., Leal-Acosta C. (2021). Salud bucal en sujetos con trastorno del espectro autista: Consideraciones para la atención odontológica. CES Odontol..

[18] Al-Oran H.M., Khuan L. (2021). Predictors of parenting stress in parents of children diagnosed with autism spectrum disorder: A scoping review. Egypt. J. Neurol. Psychiatry Neurosurg..

[19] Baker-Ericzén M.J., Brookman-Frazee L., Stahmer A. (2005). Stress Levels and Adaptability in Parents of Toddlers with and without Autism Spectrum Disorders. Res. Pract. Pers. Sev. Disabil..

[20] Di Renzo M., Guerriero V., Petrillo M., Bianchi di Castelbianco F. (2022). What is Parental Stress Connected to in Families of Children with Autism Spectrum Disorder? Implications for Parents’ Interventions. J. Fam. Issues.

[21] Galpin J., Barratt P., Ashcroft E., Greathead S., Kenny L., Pellicano E. (2018). ‘The dots just don’t join up’: Understanding the support needs of families of children on the autism spectrum. Autism.

[22] Li F., Tang Y., Li F., Fang S., Liu X., Tao M., Wu D., Jiang L. (2022). Psychological distress in parents of children with autism spectrum disorder: A cross-sectional study based on 683 mother-father dyads. J. Pediatr. Nurs..

[23] Porter N., Loveland K. (2018). An Integrative Review of Parenting Stress in Mothers of Children with Autism in Japan. Int. J. Disabil. Dev. Ed..

[24] Pozo P., Sarriá E. (2014). A global model of stress in parents of individuals with autism spectrum disorders (ASD). Ann. Psychol..

[25] Curley K., Colman R., Rushforth A., Kotera Y. (2023). Stress Reduction Interventions for Parents of Children with Autism Spectrum Disorder: A Focused Literature Review. Youth.

[26] Ni’matuzahroh, Suen M.W., Ningrum V., Widayat, Yuniardi M.S., Hasanati N., Wang J.H. (2022). The Association between Parenting Stress, Positive Reappraisal Coping, and Quality of Life in Parents with Autism Spectrum Disorder (ASD) Children: A Systematic Review. Healthcare.

[27] Ng C.S.M., Fang Y., Wang Z., Zhang M. (2021). Potential Factors of Parenting Stress in Chinese Parents of Children with Autism Spectrum Disorder: A Systematic Review. Focus Autism Other Dev. Disabl..

[28] Jolly K., Huber T. (2020). Prevalence of Anxiety and Stress in Mothers of Children with Autism Spectrum Disorder (ASD): A Review of Literature. Res. J. Ed..

[29] Dolev S., Oppenheim D., Koren-Karie N., Yirmiya N. (2009). Emotional Availability in Mother-Child Interaction: The Case of Children with Autism Spectrum Disorders. Parenting.

[30] May C., Fletcher R., Dempsey I., Newman L. (2015). Modeling Relations among Coparenting Quality, Autism-Specific Parenting Self-Efficacy, and Parenting Stress in Mothers and Fathers of Children with ASD. Parenting.

[31] Tricco A.C., Lillie E., Zarin W., O’Brien K., Colquhoun H., Kastner M., Levac D., Ng C., Pearson Sharpe J., Wilson K. (2016). A scoping review on the conduct and reporting of scoping reviews. BMC Med. Res. Methodol..

[32] AlHumaid J., Gaffar B., AlYousef Y., Alshuraim F., Alhareky M., El Tantawi M. (2020). Oral Health of Children with Autism: The Influence of Parental Attitudes and Willingness in Providing Care. Sci. World J..

[33] Alibekova R., Chan C.K., Crape B., Kadyrzhanuly K., Gusmanov A., An S., Bulekbayeva S., Akhmetzhanova Z., Ainabekova A., Yerubayev Z. (2022). Stress, anxiety and depression in parents of children with autism spectrum disorders in Kazakhstan: Prevalence and associated factors. Glob. Ment. Health.

[34] Foody C., James J., Leader G. (2014). Parenting stress, salivary biomarkers, and ambulatory blood pressure in mothers of children with Autism Spectrum Disorders. Res. Autism Spectr. Disord..

[35] Parry J.A., Newton T., Linehan C., Ryan C. (2023). Dental Visits for Autistic Children: A Qualitative Focus Group Study of Parental Perceptions. JDR Clin. Trans. Res..

[36] Permatasari I., Saskianti T., Moeharyono M. (2020). Correlation Of Mother’s Behavior In Autistic Children With DMF-T Value and Dental Care Need. Int. J. Pharm. Res..

[37] Prakash J., Das I., Bindal R., Shivu M.E., Sidhu S., Kak V., Kumar A. (2021). Parental perception of oral health-related quality of life in children with autism. An observational study. J. Family Med. Prim. Care.

[38] Verma A., Priyank H., Viswanath B., Bhagat J.K., Purbay S., Mahalakshmi V., Shivakumar S. (2022). Assessment of Parental Perceptions of Socio-Psychological Factors, Unmet Dental Needs, and Barriers to Utilise Oral Health Care in Autistic Children. Cureus.

[39] Alvesson M., Sandberg J. (2013). Constructing Research Questions: Doing Interesting Research.

[40] Omer R. (2022). Oral Health Practices and Challenges Facing Parents of Autistic Children in the Western Cape. Master’s Thesis.

[41] Zieliński G., Ginszt M., Zawadka M., Rutkowska K., Podstawka Z., Szkutnik J., Majcher P., Gawda P. (2021). The Relationship between Stress and Masticatory Muscle Activity in Female Students. J. Clin. Med..

[42] Mercante J.P.P., Oliveira A.B., Peres M.F.P., Wang Y.P., Brunoni A.R., Lotufo P.A., Benseñor I.M., Goulart A.C. (2024). Association of mental health symptoms with the migraine-tension-type headache spectrum in the Brazilian longitudinal study of adult health. J. Psychosom. Res..

[43] Enea V., Rusu D.M. (2020). Raising a Child with Autism Spectrum Disorder: A Systematic Review of the Literature Investigating Parenting Stress. J. Ment. Health Res. Intellect. Disabil..

